# Experimental *Babesia rossi* infection induces hemolytic, metabolic, and viral response pathways in the canine host

**DOI:** 10.1186/s12864-021-07889-4

**Published:** 2021-08-16

**Authors:** Rachel L. Smith, Amelia Goddard, Arun Boddapati, Steven Brooks, Johan P. Schoeman, Justin Lack, Andrew Leisewitz, Hans Ackerman

**Affiliations:** 1 Laboratory of Malaria and Vector Research, National Institute of Allergy and Infectious Disease, National Institutes of Health, Rockville, MD 20852 USA; 2 Department of Companion Animal Clinical Studies, Faculty of Veterinary Science, University of Pretoria, Private Bag X04, Onderstepoort, Pretoria, 0110 South Africa; 3 NIAID Collaborative Bioinformatics Resource (NCBR), National Institute of Allergy and Infectious Disease, National Institutes of Health, Bethesda, MD 20894 USA; 4 Advanced Biomedical Computational Science (ABCS), Frederick National Laboratory for Cancer Research, Frederick, MD 21701 USA

**Keywords:** *Babesia rossi*, Canine host, Time course RNA-seq, Host response to infection, Hemoprotozoan parasite

## Abstract

**Background:**

*Babesia rossi* is a leading cause of morbidity and mortality among the canine population of sub-Saharan Africa, but pathogenesis remains poorly understood. Previous studies of *B. rossi* infection were derived from clinical cases, in which neither the onset of infection nor the infectious inoculum was known. Here, we performed controlled *B. rossi* inoculations in canines and evaluated disease progression through clinical tests and whole blood transcriptomic profiling.

**Results:**

Two subjects were administered a low inoculum (10^4^ parasites) while three received a high (10^8^ parasites). Subjects were monitored for 8 consecutive days; anti-parasite treatment with diminazene aceturate was administered on day 4. Blood was drawn prior to inoculation as well as every experimental day for assessment of clinical parameters and transcriptomic profiles. The model recapitulated natural disease manifestations including anemia, acidosis, inflammation and behavioral changes. Rate of disease onset and clinical severity were proportional to the inoculum. To analyze the temporal dynamics of the transcriptomic host response, we sequenced mRNA extracted from whole blood drawn on days 0, 1, 3, 4, 6, and 8. Differential gene expression, hierarchical clustering, and pathway enrichment analyses identified genes and pathways involved in response to hemolysis, metabolic changes, and several arms of the immune response including innate immunity, adaptive immunity, and response to viral infection.

**Conclusions:**

This work comprehensively characterizes the clinical and transcriptomic progression of *B. rossi* infection in canines, thus establishing a large mammalian model of severe hemoprotozoal disease to facilitate the study of host-parasite biology and in which to test novel anti-disease therapeutics. The knowledge gained from the study of *B. rossi* in canines will not only improve our understanding of this emerging infectious disease threat in domestic dogs, but also provide insight into the pathobiology of human diseases caused by *Babesia* and *Plasmodium* species.

**Supplementary Information:**

The online version contains supplementary material available at 10.1186/s12864-021-07889-4.

## Background

Hemoprotozoal parasites cause significant morbidity and mortality in both humans and animals [1–6]. Parasites of the genus *Babesia* are the most common blood transfusion-transmitted pathogen in the United States and are the second most ubiquitous tick-borne pathogen in animals [7–9]. World-wide, human babesiosis is emerging as the tick vector expands its territory and encounters more people than ever before [4, 9].

*Babesia rossi*, a common parasite in wild jackals, is known to cause the most severe disease of all the *Babesia* species infecting canines [10], and is a leading cause of infectious morbidity and mortality among the susceptible domestic dog population in South Africa [11]. Over the course of several decades, observational studies of naturally-occurring *B. rossi* infections in canines have led to clinical and pathological descriptions of the disease [12, 13]. For one, clinical presentation and laboratory measurables are used to objectively classify the disease as uncomplicated or complicated. Predictors of severity and poor outcome are largely known. The host response to infection consists of pro-inflammatory cytokine storm [14]. Additionally, some individual organ systems have been described in terms of pathology; others are currently being studied [15]. Understanding the pathogenesis of severe babesiosis in a large mammal may provide insights into human severe malaria, which shares key pathophysiologic features such as hemolysis and inflammation that lead to multiple organ dysfunction [16, 17]. However, conclusions drawn from clinical case series are unreliable because the days that have elapsed from initial infection to presentation to clinic are unknown. As such, the clincal and transcriptomic time course of the disease was unable to be characterized.

To advance our understanding of the temporal dynamics of the host response to *B. rossi* infection, we performed a controlled infection with identical replicates in which both the infectious inoculum and time of infection were known. We inoculated purpose-bred canines with clinical isolates of *B. rossi* at two dose inocula and recorded clinical and laboratory measures of disease severity and progression on a daily basis. We extracted RNA from whole blood collected at six timepoints and subjected it to RNA-sequencing, differential gene expression analysis, pathway enrichment analysis, and temporal cluster analysis to identify genes and pathways that were differentially expressed as well as groups of genes that had similar expression trajectories over time. These results characterize the host response during the onset and treatment of severe babesiosis in canines and identify both known and novel pathways in the host response to severe hemoprotozoal infection.

## Results

### Clinical time course correlated with infectious inoculum

**Fig. 1:**
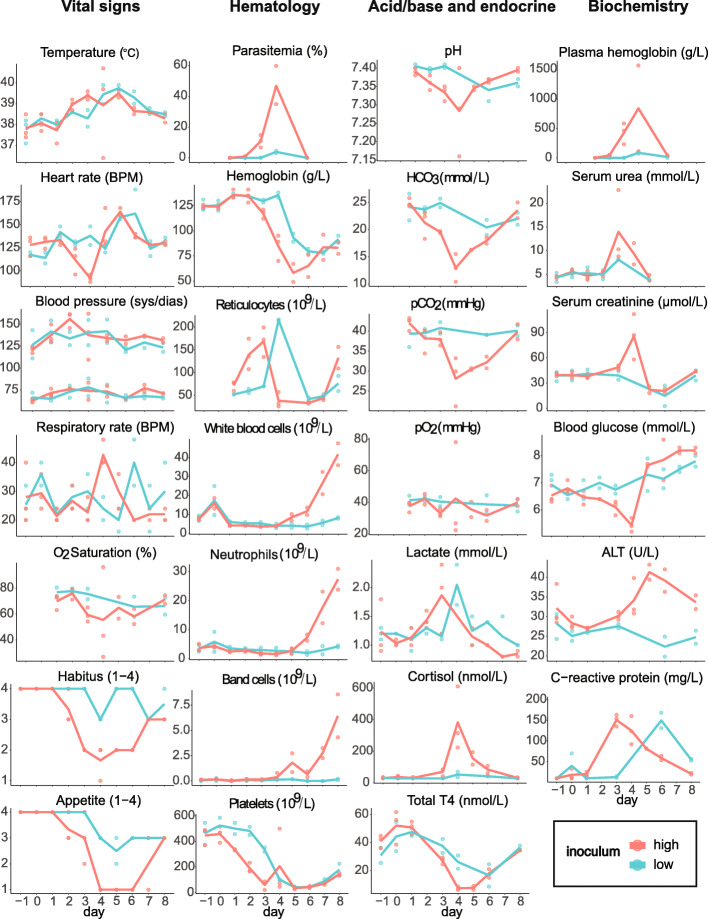
Clinical parameters recorded throughout the experiment reveal inoculum-dependent disease course and severity. Data are provided for each individual (dots) and for high and low inoculum cohort means (lines). ALT: alanine aminotransferase; BPM: beats per minute

All 5 experimentally infected subjects developed clinical disease after intravenous inoculation with 10^4^ or 10^8^ parasites. The first signs of overt clinical disease (fever, reduced habitus, and appetite loss) appeared on day 3 in the high inoculum cohort and on day 4 in the low inoculum cohort (Fig. 1). All subjects were treated on day 4 with diminazene aceturate (3.5 mg/kg intramuscularly), blood transfusion, and supportive care. One subject in the high inoculum cohort died suddenly on day 4 despite all attempts to treat it. The remaining subjects all made a full recovery and were homed as pets after completion of the experiment.

### Hematological parameters indicated severe hemolytic anemia

Parasitemia was detectable on day 2 in both the high and low inoculum cohorts. Hemoglobin concentration began to fall after day 2 in the high inoculum cohort and after day 3 in the low inoculum cohort. Plasma hemoglobin, an indicator of intravascular hemolysis, was elevated by day 3 in the high inoculum cohort. The absolute reticulocyte count was low for the degree of anemia present and peaked during peak infection (day 3 in the high inoculum and day 4 in the low inoculum cohort), then began to rise again after treatment had been administered. Both the total white blood cell count and immature neutrophil (band form) count rose steeply after day 5 in the high inoculum cohort. Severe thrombocytopenia developed by day 3 in the high inoculum cohort and by day 4 in the low inoculum cohort (Fig. 1).

### Biochemical parameters revealed impact on multiple organ systems

On days 3 and 4, signs of metabolic acidosis (low pH, low HCO_3_^−^, rising lactate) with respiratory compensation (low pCO_2_) developed in the high inoculum cohort. Serum urea and serum creatinine peaked on days 3 and 4, respectively, indicating an acute but transient decline in glomerular filtration. Serum alanine aminotransferase (ALT) peaked on day 5, indicating acute hepatocellular injury. Blood glucose fell precipitously in the high inoculum cohort and remained low until day 4 when treatment was administered. Cortisol, a stress hormone, peaked on day 4 in the high inoculum cohort. Thyroid hormone (T4) fell in both cohorts and was well below normal in the high inoculum cohort on days 4 and 5. C-reactive protein, a marker of inflammation, peaked on day 3 in the high inoculum cohort and on day 4 in the low inoculum cohort. Together, these biochemical findings describe the acute onset of a severe hemolytic and inflammatory disease affecting multiple organ systems.

### Cytokine concentrations increased during infection and recovery

**Fig. 2:**
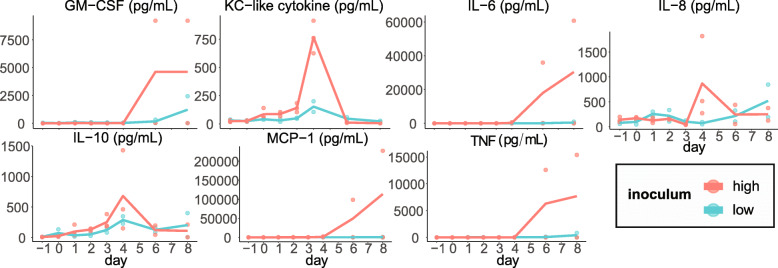
Plasma cytokine concentrations increased with parasitemia or after treatment. Data are provided for each individual (dots) and for high and low inoculum cohort means (lines)

GM-CSF (granulocyte monocyte chemotactic factor), IL-6, macrophage chemotactic protein, and TNF (tumor necrosis factor) remained low during the first 4 days following inoculation, and then rose steeply after day 4 in one subject in the high inoculum cohort. Keratinocyte chemotactic-like cytokine and IL-10 remained low during the first 3 days following inoculation, peaked in concentration on day 4, then returned to low levels following treatment. IL-8 followed this pattern in the high inoculum cohort but not the low (Fig. 2).

### Quality control assessment reported high quality sequencing data

To understand the effect of *B. rossi* inoculation on the host transcriptomic response over time, blood was drawn into Tempus tubes on experimental days 0, 1, 3, 4, 6, and 8 from each subject. High-quality RNA (mean RINe 9.4 ± 0.29) was extracted from each sample, reverse transcribed, and subjected to 100 bp paired-end next generation sequencing on a NovaSeq instrument. The number and percentage of reads mapped to the canine genome was inversely related to parasitemia. On day 3 in the high inoculum cohort and on day 4 in both cohorts (when parasitemias were greatest), the number of mapped reads (range 4.0–118.6 million) and percentage of aligned reads (mean 10.3%) was lower than on all other days, when the number of mapped reads per sample ranged from 133.6 to 260.7 million and on average 82.5% aligned to the canine reference genome (Table S1). For all samples, the range of GC content was 47–51%. The sequence data are available in the NCBI database system (https://www.ncbi.nlm.nih.gov/geo/query/acc.cgi?acc=GSE167201).

### Global expression profiles correlated with parasite density

**Fig. 3:**
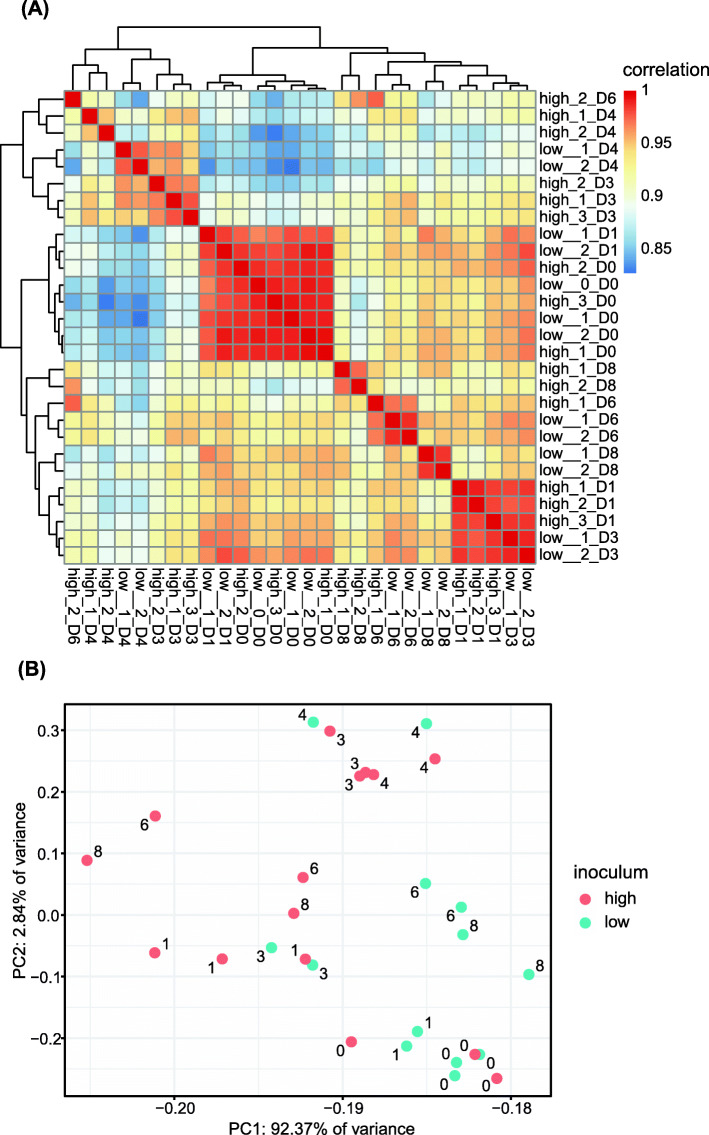
Correlation and cluster analyses of gene expression reveal primary sources of variation. **A** Heatmap showing pairwise correlation of gene expression across all samples. Raw counts were transformed using the nautral log. **B** Principal component analysis (PCA) of all samples. PCs were determined based on the natural log-transformed raw counts. Number label indicates day post-inoculation

To be included in the analysis, a gene transcript had to be present in two or more subjects in the same inoculum on the same day at 1 count per million (CPM) or greater. Eight thousand one genes passed this threshold and were used in subsequent analyses. Pairwise correlation of natural log-transformed counts for filtered host genes was high among a cluster composed of all day 0 samples and day 1 samples from the low inoculum cohort (mean *r* = 0.985; Fig. 3a). This implies a high degree of dependence of gene expression on parasitemia and a relatively low level of gene expression variation between subjects. A second cluster composed of samples from day 1 in the high inoculum cohort and day 3 in the low inoculum cohort (timepoints that coincided with the onset of parasitemia in each cohort) was also highly correlated (mean *r* = 0.984).

Principal component analysis (PCA) of natural log-transformed counts of filtered host genes revealed tight clustering of all day 0 samples along with day 1 samples from the low inoculum cohort (Fig. 3b). The first principal component, which explained 92.37% of expression variation, distinctly separated samples by inoculum during post-treatment days 6 and 8. The second principal component, which explained 2.84% of expression variation, separated samples by day of infection. Subjects that received the low inoculum underwent changes in transcriptomic profiles that were distinct for days 3, 4, 6, and 8 after infection, with day 8 returning close to day 0. Subjects that received the high inoculum underwent similar changes in transcriptomic profiles, but the changes were evident at earlier timepoints. High inoculum samples separated on PC1 on days 6 and 8, with both samples on the far left corresponding to the subject that recorded the highest levels of parasitemia. In congruency with the observed progression of clinical signs and symptoms prior to treatment, the changes in transcriptional profiles among subjects that received the low inoculum appear to lag 1–2 days behind those that received the high inoculum as shown on PC2.

### Differentially expressed genes (DEGs) were significantly upregulated throughout infection and recovery

**Fig. 4:**
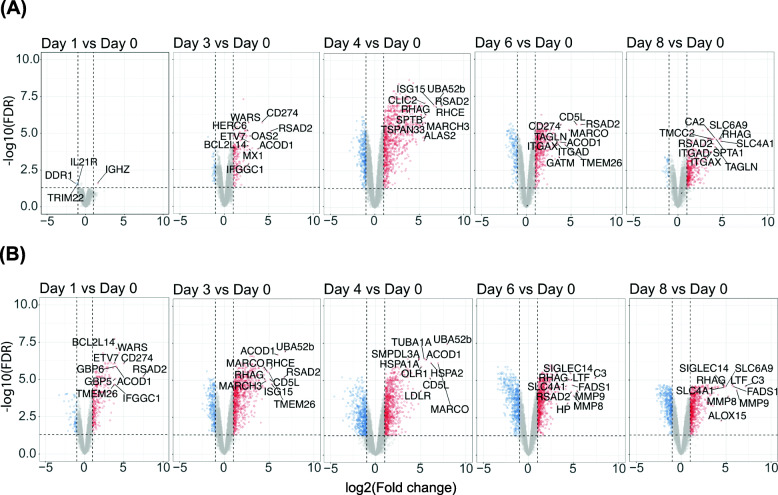
Pairwise differential gene expression for each experimental day versus day 0 in the low (**A**) and high (**B**) inoculum cohorts. Upregulated genes (log_2_(FC) > 1) are displayed in red; downregulated genes (log_2_(FC) < 1) are displayed in blue. Genes that did not meet the thresholds for significance (|log_2_(FC)| > 1; FDR < 0.05) are displayed in gray. Vertical dashed lines indicate the log_2_(FC) threshold; horizontal dashed line indicates the FDR threshold. Labeled DEGs comprise the top 20 with the highest absolute log_2_(FC) on each day in each cohort

Eight thousand one filtered genes were tested for differential expression (log_2_ fold change > |1|, FDR < 0.05), comparing expression on each experimental day versus day 0. In the low inoculum cohort, 1614 unique genes were differentially expressed on at least one experimental day (Fig. 4a). Twenty-six genes were differentially expressed on every experimental day after day 1 (on which only 4 genes were differentially expressed). In the high inoculum cohort, 2455 unique genes were differentially expressed on at least one experimental day (Fig. 4b). Fifty-two genes were differentially expressed on every experimental day after inoculation. Table 1 reports the numbers of DEGs that were up- and down-regulated on each day in each inoculum.

**Table 1:** Up- and down-regulation of DEGs in the low and high inoculum cohorts throughout the experimental course

	**Low inoculum**			**High inoculum**		
	Up-regulated	Down-regulated	Total	Up-regulated	Down-regulated	Total
**Day 1**	1 (25%)	3 (75%)	4	290 (80.6%)	70 (19.4%)	360
**Day 3**	193 (81.8%)	43 (18.2%)	236	751 (73.7%)	268 (26.3%)	1019
**Day 4**	856 (72.0%)	333 (28.0%)	1189	753 (57.0%)	568 (43.0%)	1321
**Day 6**	430 (70.6%)	179 (29.4%)	609	661 (55.5%)	529 (44.5%)	1190
**Day 8**	367 (91.3%)	35 (8.7%)	402	617 (59.6%)	419 (40.4%)	1036

Data are reported in each cell as number of DEGs (percentage of total DEGs in given inoculum on given day)

Twelve genes were differentially expressed on every experimental day after inoculation in both inoculum cohorts: *RSAD2, PSTPIP2, GCLM, OBSCN, OAS1, ISG15, DDR1, IL7R, CCR7, CHST2, APOL2,* and *TCF7* (Table S2).

### Gene ontology (GO)- and Reactome-annotated functional enrichment of DEGs

DEGs from each day in each of the high and low inoculum cohorts were separately subjected to pathway enrichment analyses using the GO and Reactome databases (Fig. S1). In the low inoculum cohort, GO pathway analysis identified 23 pathways on day 1, 221 pathways on day 3, 129 pathways on day 4, 208 pathways on day 6, and 243 pathways on day 8 to be significantly enriched. Reactome analysis identified 20 pathways on day 1, 29 pathways on day 3, 80 pathways on day 4, 68 pathways on day 6, and 56 pathways on day 8 as significantly enriched. In the high inoculum cohort, GO pathway analysis identified 281 pathways on day 1, 210 pathways on day 3, 282 pathways on day 4, 217 pathways on day 6, and 181 pathways on day 8 as significantly enriched. Reactome analysis identified 37 pathways on day 1, 48 pathways on day 3, 92 pathways on day 4, 115 pathways on day 6, and 95 pathways on day 8 as significantly enriched.

### Cluster analysis revealed temporal expression trajectories of DEGs

**Fig. 5:**
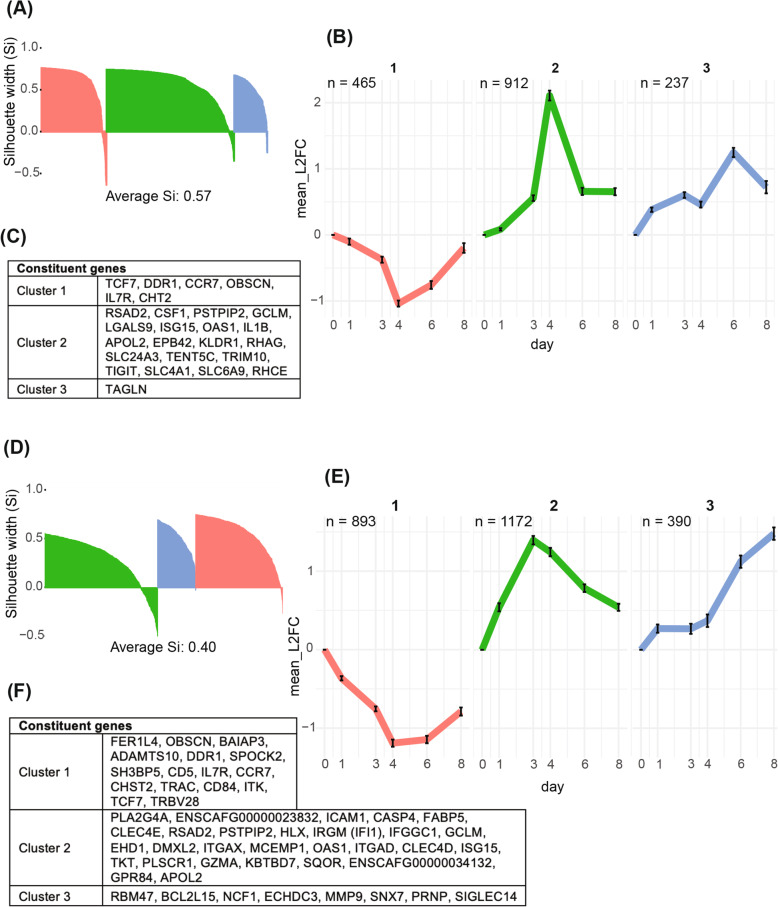
Hierarchical clustering on the log_2_(FC) of DEGs reveals three distinct temporal trajectories in each the low (**A**, **B**, **C**) and high (**D**, **E**, **F**) inoculum cohorts. **A**, **D** Silhouette plots that demonstrate how well each gene fits in its respective cluster. Silhouette width (s_i_) < 0: gene is better suited to a neighboring cluster; s_i_ > 0: gene is best suited in its assigned cluster. **B**, **E** Temporal trajectories of genes by cluster, shown as the average edgeR-calculated log_2_(FC) on each day compared to day 0 for each cluster. Cluster number is shown above each plot; color corresponds to silhouette plot color. Bars indicate 95% confidence intervals. **C**, **F** Constituent genes of each cluster that were differentially expressed on every experimental day in the high inoculum and every day except day 1 in the low inoculum cohort

Separate hierarchical cluster analyses were performed on the 1614 DEGs in the low inoculum and the 2455 DEGs in the high inoculum based on the similarity of each gene’s expression trajectory over time. Assessment of clusters using silhouette analysis [18] identified three robust clusters in each inoculum cohort, with silhouette widths (s_i_) of 0.60, 0.57, and 0.50 (weighted mean s_i_ = 0.57) and s_i_ of 0.28, 0.49, and 0.51 (weighted mean s_i_ = 0.40) in the high inoculum (Fig. 5a, d).

Each cluster had a distinct temporal trend represented as the mean (95% CI) log_2_ fold change of all genes in a cluster over time (Fig. 5b,e). In both cohorts, cluster 1 consisted of genes that were downregulated as infection progressed and rose toward baseline after treatment. In cluster 2, genes were upregulated as infection progressed and downregulated after treatment. Cluster 3 trajectories differed slightly between the cohorts. In the low inoculum, genes were upregulated during infection onset (days 1 and 3), downregulated slightly during peak infection (day 4), and upregulated after treatment (day 6) (Fig. 5b). In the high inoculum, cluster 3 consisted of genes that were upregulated during infection onset (day 1) and continued to increase expression levels after treatment (day 6) (Fig. 5e). Representative gene members of each cluster were identified as those that were differentially expressed at all time points in the high inoculum group and at all time points from day 3 onwards in the low inoculum group; these are listed in Fig. 5c and f.

## Discussion

Our understanding of the pathophysiology of canine babesiosis has, until now, been based on clinical observational studies of natural infection in a broad range of canine hosts who had been exposed to different infectious inocula at variable times in the past. To constrain these sources of clinical heterogeneity, we performed controlled inoculations at two doses in susceptible, purpose-bred laboratory beagles and characterized the infection in terms of clinical manifestations and gene expression changes over time. In doing so, we learned that experimental inoculation with wild *B. rossi* parasites consistently induces the rapid onset of symptomatic canine babesiosis. Both the rate of onset and the severity of disease were related to the size of the infectious inoculum. Disease severity, reflected through behavior, vital signs, hematology, biochemistry, acid-base status, and hormone levels, increased rapidly with the rising parasitemia and responded to treatment.

To further understand the progression of this hemoprotozoal infection which transpires entirely within the hematological system, we monitored host gene expression in circulating whole blood at frequent intervals. The results of transcriptomic analysis identified differential expression of pathways involving (1) response to hemolysis (including erythropoiesis and iron homeostasis), (2) metabolism, and (3) distinct immunological responses including innate immunity, adaptive immunity, and viral response genes. These highlight host pathways that could be targeted to slow disease progression and prevent life-threatening complications. Understanding the pathogenesis of severe babesiosis in canines can not only help us to improve the treatment of canine babesiosis, but also provide insights into the pathophysiology of human babesiosis and the related parasitic disease, severe malaria.

### Hemolysis

Anemia is a hallmark of babesiosis in humans, horses, cattle, and canines [7]. In this experimental model, massive hemolysis, as indicated by the rapid rise in plasma hemoglobin, along with inadequate erythropoiesis caused rapid and severe anemia that required transfusion to correct. After an early rise in reticulocyte counts, the reticulocytosis was suppressed as parasitemia climbed and remained inadequate for several days after treatment with an anti-parasite drug, implying that the parasite or the host response to infection was limiting erythropoiesis or blocking the release of reticulocytes from the bone marrow into circulation.

At a transcriptional level, enrichment of erythrocyte-related genes and pathways was prominent during peak parasitemia and also during recovery. During peak infection in both inoculum cohorts, oxygen transport (GO:0015671), iron ion homeostasis (GO:0055072), erythrocytes take up oxygen and release carbon dioxide (R-CFA-1247673), heme biosynthesis (R-CFA-189451), and metabolism of porphyrins (R-CFA-189445) were all among the top 10 most enriched pathways as measured by negative log FDR (Fig. S1).

Many genes with functions related to erythopoesis and heme biosynthesis were in cluster 2, which increased in expression as infection progressed and returned to baseline during recovery. This includes *KLF1*, a key transcription factor regulating erythropoiesis and hemoglobin expression [19, 20], *ALAS2*, which catalyzes the first and rate limiting step in heme synthesis in erythroid cells [21], and *UROD*, which codes an enzyme in the heme biosynthetic pathway [22]. In the same cluster, *SPTB* and *SPTA1* were differentially expressed; these genes are expressed in erythroid precursors and code for a constituent of the erythrocyte cytoskeleton [23]. Mutations in these genes are also associated with hereditary spherocytosis [24]. The rise and fall in erythroblast transcripts parallels the reticulocyte count, which peaked during peak infection (day 3 in the high inoculum and day 4 in the low inoculum cohort) and began to rise again after treatment. This association is reflected quantitatively by high correlation values between erythroblast transcript expression trajectories and the reticulocyte count (Table S3), and suggests that the changes in expression of these genes may be due to reticulocyte population dynamics.

Other genes related to hemolysis were in cluster 2. This includes *HP*, the gene that codes for haptoglobin, a protein that binds free hemoglobin from lysed red cells and is a marker for hemolysis [25]. Additionally *PRDX2*, which encodes an antioxidant enzyme that stabilizes hemoglobin under oxidative conditions, was differentially expressed in both cohorts. Mutations in this gene are strongly associated with hemolytic anemia [26]. Finally, *GCLM*, which has been shown to be essential to erythrocyte survival during oxidative stress [27] and whose deficiency is associated with hemolytic anemia [28], was also upregulated during infection.

Though there have not been extensive studies correlating gene expression with hemolysis in clinical malaria pathogenesis [29], the transcriptomic profiles reported here share many features in common with the host response to hemolytic anemia, including in sickle cell disease [20, 30–33].

### Metabolism

Both high and low dose inoculation with *B. rossi* was associated with enrichment of the Reactome pathway metabolism (R-CFA-1430728) during peak infection. In the low inoculum, long-chain fatty acid metabolic process (GO:0001676) was enriched on days 4 and 6 and cholesterol metabolic process (GO:0008203) was enriched on days 3 and 4. Both pathways were also enriched in the high inoculum on days 3 and 4 (Fig. S1).

Many lipid metabolism-related genes were in cluster 2 in both inoculum cohorts. This includes: PLA2G4A, which encodes a phospholipase involved in membrane lipid remodeling and biosynthesis of lipid mediators of the inflammatory response [34, 35]; *GPR84*, which serves as a receptor for free fatty acid [36]; *CD5L*, which encodes a regulator of lipid synthesis that in turn regulates inflammatory response mechanisms and T cell activities [37]; and *ALOX15*, which encodes a lipoxygenase that catalyzes the deoxygenation of polyunsaturated fatty acids and has known roles in red cell maturation and the inflammatory immune response [38]. Several lipid metabolism-related genes that were in cluster 2 in the low inoculum cohort were in cluster 3 in the high, indicating that they continued to increase expression levels throughout infection and recovery. This includes both *ECHDC3*, known to play a major role in fatty acid biosynthesis [39], and *FADS1*, which encodes a fatty acid desaturase [40]. Previous studies implicated the role of lipid droplets as regulators of the immune response to protozoan infection [41]. The high prevalence of lipid metabolism genes among differentially expressed genes implicates these pathways in the host response to *B. rossi* infection (Fig. 5).

Genes related to glucose metabolism were also differentially expressed throughout the infection and recovery. In the high inoculum cohort, glucose metabolic process (GO:0006006) was enriched on days 6 and 8. *GATM*, which encodes the rate-limiting step in creatine metabolism [42], was in cluster 3 in the low inoculum cohort and cluster 2 in the high. *TKT*, a key enzyme in the pentose phosphate pathway [43], was in cluster 2 in both cohorts, indicating an increase in glycolytic activity. Hypoglycemia and hyperlactatemia are a feature of both babesia and malaria infections [44, 45]. Studies in malaria show glycolysis pathway genes prominently activated early during infection in mouse models and in humans [29, 46]. Thus, canine babesiosis and human malaria appear to share features of both lipid and carbohydrate metabolic pathway activity.

### Immune response

Both the clinical and transcriptomic data indicated a robust immune response to infection in the high and low inoculum cohorts. The inflammatory nature of the disease was reflected by the inoculum-dependent changes in the fever curve, CRP level, as well as the white cell, neutrophil, and band counts – the last of which is known to predict poor outcomes [12]. While some cytokines reached a maximum at peak parasitemia (IL-8, IL-10, KC-like) others rose only after the infection was treated (TNF, IL-6, MCP-1). Multiple immune pathways were differentially regulated in response to both high and low inocula. Defense response (GO:0006952) was enriched in the high inoculum on day 1 and both inoculum cohorts on day 3. Immune system/response (R-CFA-168256; GO:0006955) was enriched in the high inoculum on days 1 and 8, and both inoculum cohorts on days 3–6. Innate immune system/response (R-CFA-168249; GO:0006955) was enriched on every experimental day in the high inoculum cohort. Additionally, cytokine signaling in immune system (R-CFA-1280215) was enriched during infection onset in both inoculum cohorts (day 1 in the high and day 3 in the low inoculum) These pathways are composed of hundreds of constituent genes, which primarily followed cluster 2 trajectories. These pathways reflect the differential expression of specific genes involved in innate immunity, adaptive immunity, and viral response (Figs. 4, S1).

### Innate immunity

Genes associated with positive regulation of interleukin-1 beta (IL-1β) production (GO:0032731) were in cluster 2. This includes: *IL1B*, which encodes the pro-inflammatory cytokine [47]; *PSTPIP2*, which encodes a protein that regulates IL-1β [48, 49]; *CASP4*, which regulates IL-1β synthesis in macrophages [50]; and *SIGLEC14*, which encodes a receptor that enhances inflammasome activation and macrophage IL-1β release. Furthermore, genes associated with NF-kB signaling were also constituents cluster 2, including *FABP5*, *ACOD1*, and *TNFAIP2*. *FABP5* encodes a fatty acid binding-protein that promotes the activation of NF-kB signaling [51]. *ACOD1* serves a multifaceted role in the innate immune response to pathogen infection (bacteria and viruses) and in cytokine signaling (TNF and interferons), and is associated with both Toll-like receptor and NF-kB signaling pathways [52]. Expression of *TNFAIP2* is upregulated via the NF-kB pathway and mediates the inflammatory response [53, 54]. Studies of malaria infection in humans also implicate the role of IL-1β signaling and NF-kB pathways in the innate immune response to infection [55, 56]. Thus, *Babesia* and *Plasmodium* induce similar innate immune responses across different host species.

Pathways associated with the production and regulation of interferon-gamma (GO:0032649) were significantly enriched in the host response to *B. rossi* infection, especially during infection onset (Fig. S1). IFN-γ signaling-related genes, including *IRGM* (*IFI1*), *IFGGC1*, *SPP1*, and *APOL2*, were in cluster 2 in both inoculum cohorts. *IRGM* plays a key role in regulating IFN-γ-induced host defense to protozoa [57], while *IFGGC1* is a member of the IFN-γ-inducible GTPases that are involved in immune response against pathogens [58]. *SPP1* codes for a cytokine known to upregulate IFN-γ production [59], and *APOL2* which mitigates the cytotoxic effects of IFN-γ [60]. IFN-γ is a major pro-inflammatory cytokine that has both direct antiparasitic activity and immunoregulatory roles in the response to parasitic infection [61]. IFN-γ has a role in protective immunity against infection by *B. microti* in mice [62] and is involved in the host immune response to malaria in both mice and humans [29, 55, 56]. Our findings suggest IFN-γ has a similar role in protective immunity against *B. rossi* infection in canines.

### Adaptive immunity

Genes that code the MHC class II protein complex, namely *DLA-DOA* and *DLA-DOB* [63], were in cluster 1, indicating that they were significantly downregulated in both inoculum cohorts. In contrast, MHC class I genes *DLA-12, DLA-64*, and *DLA-88* were upregulated during infection (though they did not meet the log_2_ fold change threshold for significance, excepting the low inoculum cluster 2 gene *DLA-12*). Additionally, *GZMA* and *PRDX2*, genes specifically expressed by CD8+ T cells and associated with their activity [64, 65], were cluster 2 DEGs in both cohorts.

CD8+ and CD4+ T cells, whose T-cell receptors recognize antigens presented by MHC-I and MHC-II respectively, have been previously implicated in protective immunity against *B. microti* infection in mice and *B. bovis* infection in cattle. Previous studies in mice and humans suggest that both MHC-I and MHC-II play a role in the host immune response to malaria infection [29, 56]. However, recent studies in human cerebral malaria suggest that CD8+ T cells migrate to and sequester in the brain and contribute to mortality [66, 67]. Our data reveal downregulation of genes associated with MHC class II CD4+ T cells and potential induction of MHC class I CD8+ T cells in the host response to *B. rossi*, raising the question of what cells are presenting antigens and whether the CD8+ and CD4+ responses are immunopathologic or contribute to parasite killing and adaptive immunity.

### Viral response

Interestingly, many viral response genes were differentially regulated during *B. rossi* infection, particularly during infection onset. On day 1 in the high inoculum cohort, pathways including positive regulation of defense response to virus by host (GO:0002230), antiviral mechanisms by IFN-stimulated genes (R-CFA-1169410), and ISG15 antiviral mechanism (R-CFA-1169408) were enriched. The latter two pathways were also enriched on day 3 in the low inoculum cohort, along with response to interferon-alpha (GO:0035455) and cellular response to interferon-beta (GO:0035458). (Fig. S1).

This is also reflected in the significant upregulation of constituent viral response genes throughout the infection in both inoculum groups. All of the following genes were identified as cluster 2 DEGs: *RSAD2, ISG15*, *DDX58, DDX60, OAS1*, *OAS2, OASL1, MX1*, *PLSCR1*, *ICAM1*, *TRIM10*, *KLRD1*, *LGALS9*, and *LTF*. In general these genes are related to type I interferon (IFN-I) antiviral signalling and have been associated with a range of viruses including influenza, west Nile virus, hepatitis C, and dengue virus [68–82]. Interestingly, previous studies in malaria and leishmaniasis models suggest that type I interferons may also suppress anti-parasitic immunity [83–86]. CD8+ T cells are also associated with antiviral activity [64, 65, 87, 88]. Together, these transcriptomic data implicate the role of viral response genes and pathways in the host immune response to *B. rossi* infection.

In the context of *Plasmodium* infection, parasite DNA and RNA have been shown to bind to receptors that activate downstream production of IFN-Is. For one, toll-like receptor 9 (TLR9) recognizes parasite DNA [89, 90]. Cyclic GMP-AMP synthase (cGAS) also binds to parasite DNA in the cytoplasm and catalyzes the synthesis of cyclic guanosine monophosphate-adenosine monophosphate (cGAMP), which acts as a second messenger to activate the downstream signal adaptor, stimulator of interferon genes (STING) [89, 91, 92]. Furthermore, major cytosolic receptor for sensing malaria parasite RNA is melanoma differentiation-associated protein 5 (MDA5) [92, 93]. Parasite RNA also possibly stimulates the retinoic acid-inducible gene I (RIG-I) pathway [94], though this has yet to be confirmed in vivo [89]. It is likely that *Babesia* parasite DNA and RNA similarly activate receptors that activate downstream production of IFN-Is. Of note, it is possible that different sensors are activated by parasite DNA and RNA at different developmental stages, and IFN-I subtypes (IFN-α and IFN-β) have different biological and immunological roles in viral infections [89]. Future experiments will explore the mechanistic roles of IFN-Is in *B. rossi* infection.

### Limitations

The inoculum of 10^9^ parasites resulted in an acute and severe disease course. This rapid disease course may be more representative of transfusion-associated babesiosis than natural tick-transmitted infections which would evolve more slowly. Future experiments will utilize a lower infectious inoculum, which may be more representative of natural infections. *B. bovis* and *Plasmodium falciparum* are known to cytoadhere to the vascular endothelium in severe babesiosis and malaria [95–98], respectively, while *P. vivax* and *P. knowlesi* can cause severe disease without evidence of cytoadherence [99–102]. The available evidence suggests that *B. rossi* causes severe disease without cytoadherence to vascular endothelium [103–105]. The low-mapped reads on day 4 in the high and low inoculum cohorts and day 3 in the high appear to be associated with the high levels of parasitemia recorded on those days. The lower percentage of mapped reads indicates lower overall sequencing accuracy and the potential presence of contaminating DNA, thus increasing the likelihood of spurious results on these days. Additionally, one animal in the high inoculum died on the morning of day 4, which may have affected the expression means reported on days 4, 6, and 8. Transcriptomic and clinical results reported on days 6 and 8 may have been further affected by the administration of an anti-parasite drug diminazene and blood transfusions on day 4. While the primary effect of the drug is to reduce parasitemia, it may also have a weak anti-inflammatory effect [106]. Furthermore, both cohorts were small (low *n* = 2; high *n* = 3); however, there was a high degree of consistency between subjects of the same cohort with regard to clinical observations and transcriptomic data.

It is likely that cellular population dynamics account for many of the changes in gene expression observed, as exemplified by the correlation of reticulocyte count with erythroblast transcripts. When pure cellular reference populations are available, cell-type deconvolution can be applied to understand the relative contributions of changes in gene expression versus changes in cell abundance. Another approach would be to measure gene expression at the single cell level. Either of these techniques could be employed to identify transcripts that changed in expression levels within a cell population as opposed to transcripts whose changes in expression were associated with changing cellular populations.

## Conclusions

This study is the first to describe canine host gene expression induced by *B. rossi* infection. The rate of onset and disease severity were inoculum-dependent, as shown by clinical data including vital signs, hematological parameters, parasitemia, acid-base chemistry, and biochemical markers. The temporal dynamics of differential gene expression and pathway enrichment implicate response to parasite burden, hemolysis, induction of lipid metabolism, and a robust role of different immunological pathways including those related to innate immunity, adaptive immunity, and response to viral infection. These findings offer a fundamental understanding of the host transcriptomic response to *B. rossi*, which can serve as a model of human hemolytic diseases caused by intracellular parasites, such as babesiosis and malaria.

## Methods

### Animals

All methods were carried out in accordance with relevant guidelines and regulations. Six purpose bred male beagles were born and raised in the Onderstepoort Veterinary Animal Research Unit. The animals were all microchipped, vaccinated and dewormed according to international guidelines and neutered at 6 months of age. Stringent ectoparasite control was applied by means of the isolation of the housing facility and 3 monthly dosing with an isoxazoline compound. All dogs were proven to be free of any blood borne parasites by PCR and reverse line blot. All dogs were housed communally in a play enriched environment and socialized with animal care givers each day. They were trained through a food reward system to become accustomed to being held for blood collection and blood pressure monitoring on a daily basis for several months before the beginning of the experiment.

### Creation of a Babesia rossi cryopreservate

Three to five milliliters of whole venous blood were collected into EDTA vacutainer tubes from dogs naturally infected with *Babesia rossi* and presented for veterinary care at the Onderstepoort Veterinary Academic Hospital and cryopreserved in dimethylsuphoxide. All samples collected this way were subjected to PCR and reverse line blot to confirm a mono-infection with *B. rossi*. One of these samples was later used to infect a splenectomized Beagle dog to raise the infectious inoculum required for the infection of the experimental dogs.

### Experimental infection

Animals were cared for in compliance with the ARRIVE (Animal Research: Reporting of In Vivo Experiments) guidelines on an experimental protocol approved by the University of Pretoria Animal Ethics Committee (V003–18). One of the 6 Beagles was splenectomized at 7 months of age, allowed to recover for 3 weeks and then infected intravenously with one of the wild-type *B. rossi* cryopreserved samples by intravenous injection. This canine was monitored closely, and once a patent parasitemia of ~ 2% was detected on blood smear of central venous blood, the parasitaemia was accurately quantified, and 10 mL of blood was collected into acid citrate dextrose solution and diluted with a culture medium used for in-vitro babesia culture to create infectious inocula, two doses of 10^4^ parasites and three of 10^8^ parasites. The splenectomized dog was immediately treated and made a full recovery without becoming obviously ill. These inocula were immediately injected intravenously into two canines that received the low infectious dose and three that received the high infectious dose. These five canines were then followed carefully through frequent physical observation and daily blood collections.

### Biospecimen collection

Blood was collected daily into EDTA tubes for complete blood counts and the harvesting of plasma for cytokine determination, clot tubes for serum biochemistry, heparinized blood collected anaerobically for venous blood gas, lactate and glucose determination, citrated tubes for coagulation studies and RNALater tubes (Tempus), for the transcriptomic study reported here. Baseline data was generated twice at a 2-week interval before any infections took place to generate a baseline.

### Clinical and laboratory data collection

Clinical examinations and all blood samples were collected from the jugular vein with 21G vacutainer needles (Precision Glide™, UK) between 8:00 AM and 10:00 AM daily. The following observational data were collected once every day for each experimental subject: habitus and mental status (scored 1–4: 1-collapsed, 2-very weak, just able to stand, 3-able to stand but depressed, 4-normal interaction); appetite (scored 1–4: 1-no interest in food, 2- slight interest with minimal ingestion, 3-partial ingestion of a full meal, 4-normal ingestion of the whole meal); rectal temperature; pulse (palpated on the femoral artery); respiratory rate counted by physical observation; mucous membrane color and capillary refill time; thoracic auscultation; abdominal palpation; stained blood smear examination; blood pressure.

Haematology was performed on a venous sample collected into an EDTA Vacutainer Brand Tubes (Beckton Dickinson Vacutainer Systems, UK) from the jugular and was run on an ADVIA 2120 (Siemens, Munich, Germany). within an hour of collection A differential count was performed manually by an experienced laboratory technologist. Siemens, Germany) within 1 h of blood collection. The remaining volume of blood was then centrifuged and aliquoted within 30 min of collection for storage of EDTA plasma at -80 °C.

Serum biochemistry samples were collected in Serum Vacutainer Brand Tubes (Beckton Dickinson Vacutainer Systems, UK), and the CRP (using canine specific immunoturbidimetric CRP method), albumin (using a colorimetric assay with bromocresal green) were determined. Glucose (using the point of care AlphaTRAK 2) and the automated hexokinase method was run on an automated chemistry analyser (Cobas Integra© 400Plus). This sample was allowed to clot for 10 min, samples were run and the remaining serum volumes were aliquoted within 30 min of collection for storage at -80 °C.

Blood gas samples were collected anaerobically into a commercially prepared heparinized syringe (BD A-Line, arterial blood collection syringe, Becton, Dickinson and Company, UK) using a 21G needle from the jugular. Lactate was obtained directly from a fresh blood sample using a Lactate Pro 2 hand held lactate reader and from the venous blood gas analysis, analyzed within 20 min (Rapidpoint 405, Seimens).

The cytokines measured included: GM-CSF, IFN-γ, IL-2, IL-6, IL-7, IL-8, IL-15, IL-10, IL-18, TNF-α, IP 10, KC and MCP-1. Concentrations were determined in duplicate by fluorescent-coded magnetic beads (MagPlex-C; MILLIPLEX. MAP Kit, Canine Cytokine Magnetic Bead Panel, 96-Well Plate Assay, CCYTO-90 K, Millipore, Billerica, MA), based on the Luminex xMAP technology (Luminex 200, Luminex Corporation, Austin, TX). Two quality controls were included in the plate as internal quality controls. The assay was performed according to the manufacturer’s instructions.

Blood pressure was measured using the Vet HDO® MDPro and the HDO management software. The following protocol for blood pressure measurement was standardized: The environment was isolated, quiet and away from other animals. The same environment was used every day and the canines were trained and conditioned for 4 weeks prior to the onset of the experiment. No sedation was used, and the canines were allowed 5–10 min to relax and become accustomed to the environment. Each subject was gently restrained in right lateral recumbency. The cuff width was approximately 40% of the circumference of the cuff site and the cuff size was recorded at every measurement. The cuff was placed around the tail base. All blood pressure measurements were performed by the same person every day. The canines were calm and relatively motionless as they were all well-conditioned to the process prior to the start of the experimental period. The first measurement was discarded. Five consecutive and consistent (< 20% variability in systolic values) were recorded and the average of the values was calculated to obtain the blood pressure measurement.

### RNA isolation

Blood from each subject was collected into Tempus Blood RNA Tubes on days 0, 1, 3, 4, 6, and 8 and subsequently stored at -20 °C. Samples were shipped on dry ice to the United States, where they remained at -20 °C prior to RNA extraction. RNA was isolated using Qiagen RNeasy Mini Kit. RNA quality was controlled on a Bioanalyzer Instrument. All RNA samples had an RNA Integrity Number (RIN) of ≥9.7. Two independent biological replicates were selected for each time point for subsequent RNA sequencing.

### RNAseq library preparation and sequencing

PolyA-selected mRNA libraries were generated and all samples were indexed and pooled before sequencing in a single S1 flowcell on an Illumina NovaSeq.

### Data analysis

Reads were trimmed and mapped to the canine reference genome using the Center for Cancer Research Collaborative Bioinformatics Resource (CCBR) Pipeliner. Pipeliner provides open access to some of the next-generation sequencing data analysis pipelines used by CCBR. It allows users to select a set of genomes and data files, then processes them through a sequence of programs to produce the desired output (i.e. raw counts matrix, QC report, differentially expressed genes, etc.). Specifically, Pipeliner employs the reference-mapping tool STAR, which is a widely used and accepted tool and allows for mismatches and sequence divergence well beyond that expected amongst canine breeds [107]. Once configured, the pipeline is executed on the NIH high performance computing cluster Biowulf. Pipeliner is publicly available on GitHub [https://github.com/CCBR/Pipeliner].

Raw gene counts were converted to counts per million (CPM). Genes with fewer than 1 CPM in two or more treatment group samples (inoculum + day) were excluded from analysis. Differential expression analysis was run for each experimental day compared to day 0 in each inoculum cohort using the edgeR package (3.30.3), and *p*-values were corrected using the Benjamini-Hochberg (BH) method. Genes with FDR < 0.05 and log_2_ fold change > |1| were considered significantly differentially expressed. Gene Ontology (GO)-annotated pathway enrichment analysis was performed using the topGO package (2.40.0); package authors discourage multiple testing correction as the p-values returned are already conservative [108]. Reactome-annotated pathway enrichment analysis was performed using the biomaRt (2.44.0) and reactome.db (1.70.0) packages, with significance assigned according to a hypergeometric distribution and p-values corrected using the BH method. Hierarchical cluster analyses were performed separately for each inoculum cohort, and included all genes that were differentially expressed on at least one experimental day. The Pearson correlation between genes was calculated using the edgeR-calculated log_2_ fold change across experimental days. The Euclidean distance between those correlation values was then calculated and clustering was performed using Ward’s method. Cluster robustness was assessed using silhouette analysis as part of the factoextra R package (1.0.7). To visualize cluster temporal trajectories, the mean edgeR-calculated log_2_ fold change on each day within each cluster was plotted along with 95% confidence intervals. Data analysis was conducted in R version 4.0.0.

## Supplementary Information

**Additional file 1: Table S1.** A summary of MultiQC data. Shown are the number of reads mapped (in millions), percent of these reads aligned to the canine reference genome, and percent GC content of these reads. Samples corresponding to low inoculum day 4 and high inoculum days 3 and 4 were not included in the mean.

**Additional file 2: Table S2.** DEGs on every day in both inoculum cohorts. Genes that were differentially expressed on every experimental day in both cohorts (except day 1 in the low inoculum) are shown, along with their corresponding cluster.

**Additional file 3: Table S3.** Pearson correlation values of erythroblast transcript trajectory with reticulocyte count. The expression level of each gene (in CPM) was correlated with the reticulocyte count (109/L) on each day. This allowed for correlation based on expression trajectory through time.

**Additional file 4: Fig. S1.** Top 10 Gene Ontology (GO)- and Reactome-annoted pathways by FDR on each day in each inoculum cohort (A – low; B – high). x-axis: percentage of genes in pathway that were identified as differentially expressed on the respective day; y-axis: pathway name; point color: absolute number of genes associated with the pathway that were identified as differentially expressed on the corresponding day. Note that color does not indiciate up- or down-reulgation of genes in pathway.

## Data Availability

The datasets supporting the conclusions of this article are available on the NCBI Gene Expression Omnibus (GEO) database as Series GSEI67201 [https://www.ncbi.nlm.nih.gov/geo/query/acc.cgi?acc=GSE167201]. The code generated for this analysis is available at the GitHub repository Babesia_RNAseq [https://github.com/rlsmith1/Babesia_RNAseq].

## Abbreviations

ALT: Alanine aminotransferase
CPM: Counts per million
DEGs: Differentially expressed genes
FC: Fold change
FDR: False discovery rate
GM-CSF: Granulocyte monocyte chemotactic factor
GO: Gene Ontology
NCBI: National Center for Biotechnology Information
PCA: Principal Components Analysis
RIN: RNA integrity number
TNF: Tumor necrosis factor
