# Supplementary material for: Experimental Babesia rossi infection induces hemolytic, metabolic, and viral response pathways in the canine host
Source: BMC Genomics. 2021 Aug 16;22:619. doi: 10.1186/s12864-021-07889-4 (PMC8369750; doi:10.1186/s12864-021-07889-4)
Supplement: Supplementary file 1 — Additional file 1: Table S1. A summary of MultiQC data. Shown are the number of reads mapped (in millions), percent of these reads aligned to the canine reference genome, and percent GC content of these reads. Samples corresponding to low inoculum day 4 and high inoculum days 3 and 4 were not included in the mean. [file 12864_2021_7889_MOESM1_ESM.pdf]

Sample	M Reads Mapped	% Aligned	% GC
low_0_D0	213.4	85.5	50
low_1_D0	143	76.1	48
low_2_D0	166.6	83.4	50
high_1_D0	164.5	81.8	50
high_2_D0	210.2	84.7	50
high_3_D0	182.6	84	50
low_1_D1	176	82.3	50
low_2_D1	198	83.5	50
high_1_D1	175.3	80.8	50
high_2_D1	212.1	78.7	50
high_3_D1	174.1	80.3	50
low_1_D3	227	79.2	51
low_2_D3	194.9	78	51
high_1_D3	47	12.7	48
high_2_D3	30.1	7.4	47
high_3_D3	26.3	7.1	47
low_1_D4	118.6	25.9	49
low_2_D4	68	16	48
high_1_D4	7	2.2	47
high_2_D4	4	1.3	47
low_1_D6	133.6	81.7	49
low_2_D6	167.8	82.8	50
high_1_D6	135.5	81.6	48
high_2_D6	137.8	82.4	49
low_1_D8	168.1	84.6	50
low_2_D8	260.7	87.4	52
high_1_D8	176.8	86.8	51
high_2_D8	202.1	86.3	51
**Mean =**	**181.9**	**82.5**	**50**

Table S1. A summary of MultiQC data.
Shown are the number of reads mapped (in millions), percent of these reads aligned to the canine reference genome, and percent GC content of these reads. Samples corresponding to low inoculum day 4 and high inoculum days 3 and 4 were not included in the mean.
