# Supplementary material for: Experimental Babesia rossi infection induces hemolytic, metabolic, and viral response pathways in the canine host
Source: BMC Genomics. 2021 Aug 16;22:619. doi: 10.1186/s12864-021-07889-4 (PMC8369750; doi:10.1186/s12864-021-07889-4)
Supplement: Supplementary file 2 — Additional file 2: Table S2. DEGs on every day in both inoculum cohorts. Genes that were differentially expressed on every experimental day in both cohorts (except day 1 in the low inoculum) are shown, along with their corresponding cluster. [file 12864_2021_7889_MOESM2_ESM.pdf]

**Ensemble gene ID**	**Gene symbol**	**Cluster**
ENSCAFG00000003293	RSAD2	2
ENSCAFG00000017656	PSTPIP2	2
ENSCAFG00000020126	GCLM	2
ENSCAFG00000001161	OBSCN	1
ENSCAFG00000023556	OAS1	2
ENSCAFG00000019348	ISG15	2
ENSCAFG00000000448	DDR1	1
ENSCAFG00000018752	IL7R	1
ENSCAFG00000030300	CCR7	1
ENSCAFG00000008005	CHST2	1
ENSCAFG00000029830	APOL2	2
ENSCAFG00000000990	TCF7	1

Table S2. DEGs on every day in both inoculum cohorts.
Genes that were differentially expressed on every experimental day in both cohorts (except day 1 in the low inoculum) are shown, along with their corresponding cluster.
