# Supplementary material for: Experimental Babesia rossi infection induces hemolytic, metabolic, and viral response pathways in the canine host
Source: BMC Genomics. 2021 Aug 16;22:619. doi: 10.1186/s12864-021-07889-4 (PMC8369750; doi:10.1186/s12864-021-07889-4)
Supplement: Supplementary file 3 — Additional file 3: Table S3. Pearson correlation values of erythroblast transcript trajectory with reticulocyte count. The expression level of each gene (in CPM) was correlated with the reticulocyte count (109/L) on each day. This allowed for correlation based on expression trajectory through time. [file 12864_2021_7889_MOESM3_ESM.pdf]

Ensembl gene ID	Gene symbol	Correlation with reticulocyte count
ENSCAFG00000004646	UROD	0.82
ENSCAFG00000011638	SPTA1	0.07
ENSCAFG00000014308	ALAS2	0.78
ENSCAFG00000016120	SPTB	0.7
ENSCAFG00000017111	KLF1	0.83

Table S3. Pearson correlation values of erythroblast transcript trajectory with reticulocyte count.
The expression level of each gene (in CPM) was correlated with the reticulocyte count (10^9/L) on each day. This allowed for correlation based on expression trajectory through time.
