# Supplementary material for: Experimental Babesia rossi infection induces hemolytic, metabolic, and viral response pathways in the canine host
Source: BMC Genomics. 2021 Aug 16;22:619. doi: 10.1186/s12864-021-07889-4 (PMC8369750; doi:10.1186/s12864-021-07889-4)
Supplement: Supplementary file 4 — Additional file 4: Fig. S1. Top 10 Gene Ontology (GO)- and Reactome-annoted pathways by FDR on each day in each inoculum cohort (A – low; B – high). x-axis: percentage of genes in pathway that were identified as differentially expressed on the respective day; y-axis: pathway name; point color: absolute number of genes associated with the pathway that were identified as differentially expressed on the corresponding day. Note that color does not indiciate up- or down-reulgation of genes in pathway. [file 12864_2021_7889_MOESM4_ESM.pdf]

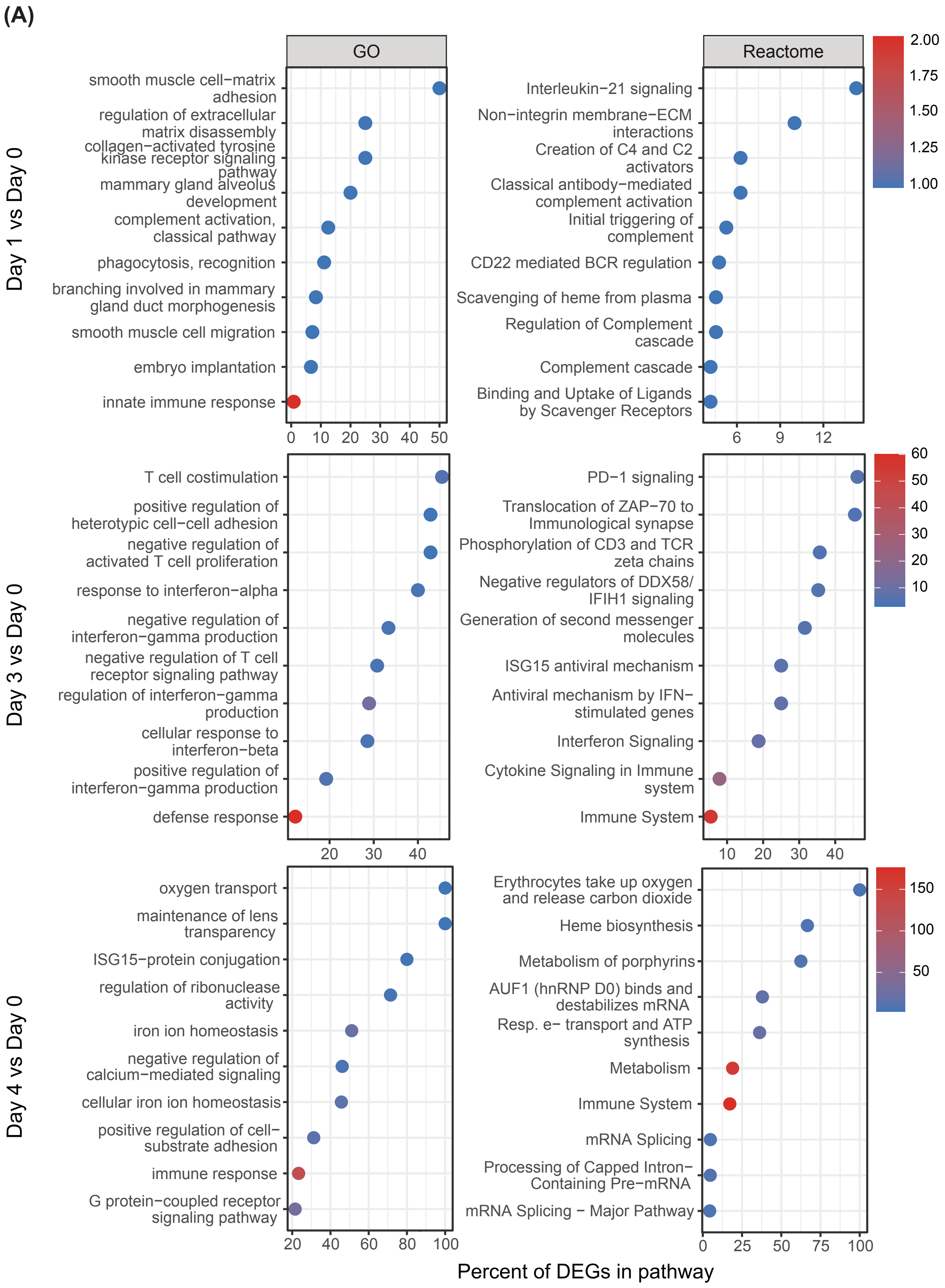

(A)
GO
Reactome
Day 1 vs Day 0
smooth muscle cell−matrix adhesion
regulation of extracellular matrix disassembly
collagen−activated tyrosine kinase receptor signaling pathway
mammary gland alveolus development
complement activation, classical pathway
phagocytosis, recognition
branching involved in mammary gland duct morphogenesis
smooth muscle cell migration
embryo implantation
innate immune response
0 10 20 30 40 50
Interleukin−21 signaling
Non−integrin membrane−ECM interactions
Creation of C4 and C2 activators
Classical antibody−mediated complement activation
Initial triggering of complement
CD22 mediated BCR regulation
Scavenging of heme from plasma
Regulation of Complement cascade
Complement cascade
Binding and Uptake of Ligands by Scavenger Receptors
6 9 12
2.00 1.75 1.50 1.25 1.00
Day 3 vs Day 0
T cell costimulation
positive regulation of heterotypic cell−cell adhesion
negative regulation of activated T cell proliferation
response to interferon−alpha
negative regulation of interferon−gamma production
negative regulation of T cell receptor signaling pathway
regulation of interferon−gamma production
cellular response to interferon−beta
positive regulation of interferon−gamma production
defense response
20 30 40
PD−1 signaling
Translocation of ZAP−70 to Immunological synapse
Phosphorylation of CD3 and TCR zeta chains
Negative regulators of DDX58/ IFIH1 signaling
Generation of second messenger molecules
ISG15 antiviral mechanism
Antiviral mechanism by IFN− stimulated genes
Interferon Signaling
Cytokine Signaling in Immune system
Immune System
10 20 30 40
60 50 40 30 20 10
Day 4 vs Day 0
oxygen transport
maintenance of lens transparency
ISG15−protein conjugation
regulation of ribonuclease activity
iron ion homeostasis
negative regulation of calcium−mediated signaling
cellular iron ion homeostasis
positive regulation of cell− substrate adhesion
immune response
G protein−coupled receptor signaling pathway
20 40 60 80 100
Erythrocytes take up oxygen and release carbon dioxide
Heme biosynthesis
Metabolism of porphyrins
AUF1 (hnRNP D0) binds and destabilizes mRNA
Resp. e− transport and ATP synthesis
Metabolism
Immune System
mRNA Splicing
Processing of Capped Intron− Containing Pre−mRNA
mRNA Splicing − Major Pathway
0 25 50 75 100
150 100 50
Percent of DEGs in pathway

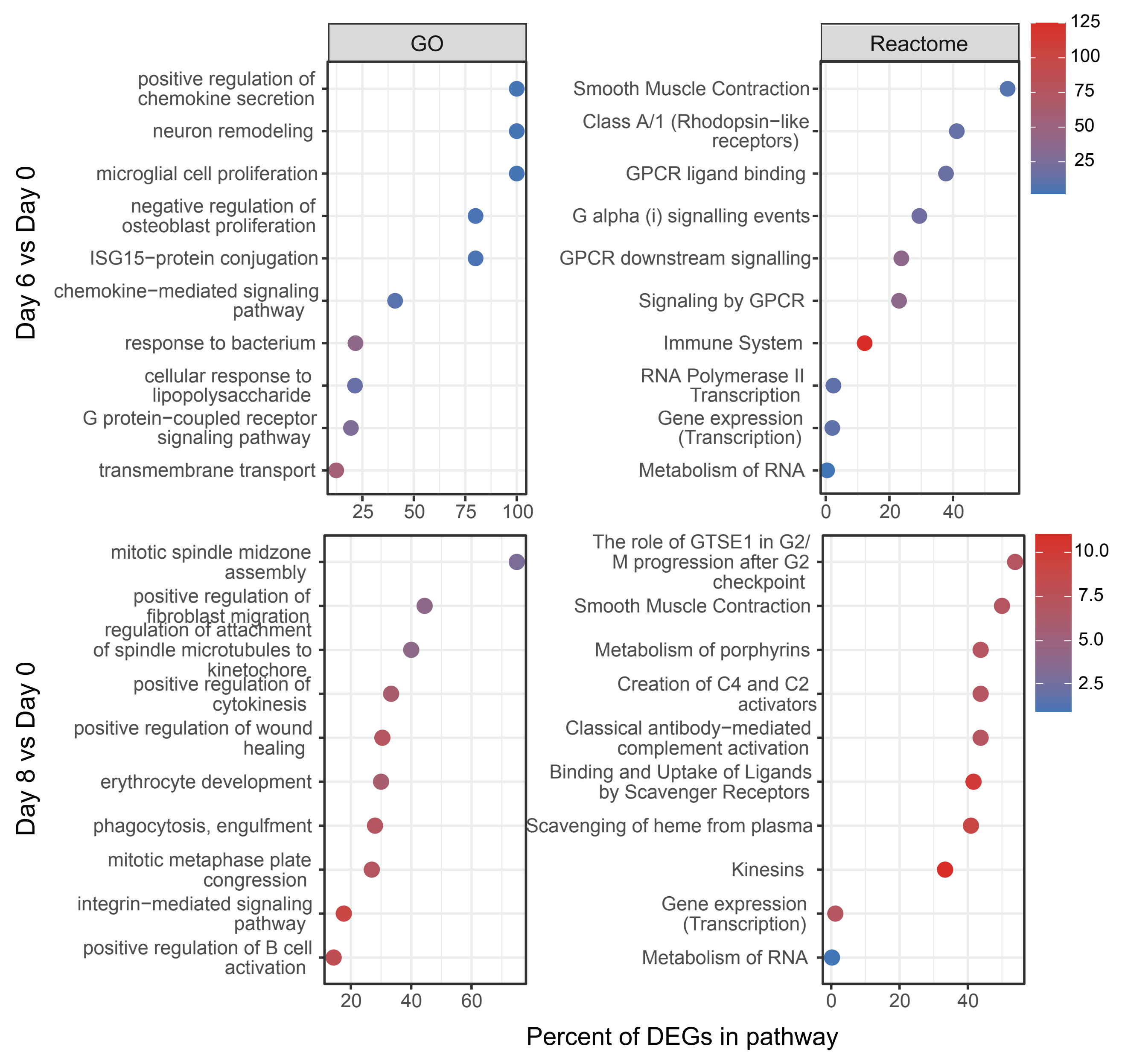
GO
Reactome
Day 6 vs Day 0
positive regulation of chemokine secretion
neuron remodeling
microglial cell proliferation
negative regulation of osteoblast proliferation
ISG15−protein conjugation
chemokine−mediated signaling pathway
response to bacterium
cellular response to lipopolysaccharide
G protein−coupled receptor signaling pathway
transmembrane transport
25
50
75
100
Smooth Muscle Contraction
Class A/1 (Rhodopsin−like receptors)
GPCR ligand binding
G alpha (i) signalling events
GPCR downstream signalling
Signaling by GPCR
Immune System
RNA Polymerase II Transcription
Gene expression (Transcription)
Metabolism of RNA
0
20
40
125
100
75
50
25
Day 8 vs Day 0
mitotic spindle midzone assembly
positive regulation of fibroblast migration
regulation of attachment of spindle microtubules to kinetochore
positive regulation of cytokinesis
positive regulation of wound healing
erythrocyte development
phagocytosis, engulfment
mitotic metaphase plate congression
integrin−mediated signaling pathway
positive regulation of B cell activation
20
40
60
The role of GTSE1 in G2/M progression after G2 checkpoint
Smooth Muscle Contraction
Metabolism of porphyrins
Creation of C4 and C2 activators
Classical antibody−mediated complement activation
Binding and Uptake of Ligands by Scavenger Receptors
Scavenging of heme from plasma
Kinesins
Gene expression (Transcription)
Metabolism of RNA
0
20
40
10.0
7.5
5.0
2.5
Percent of DEGs in pathway

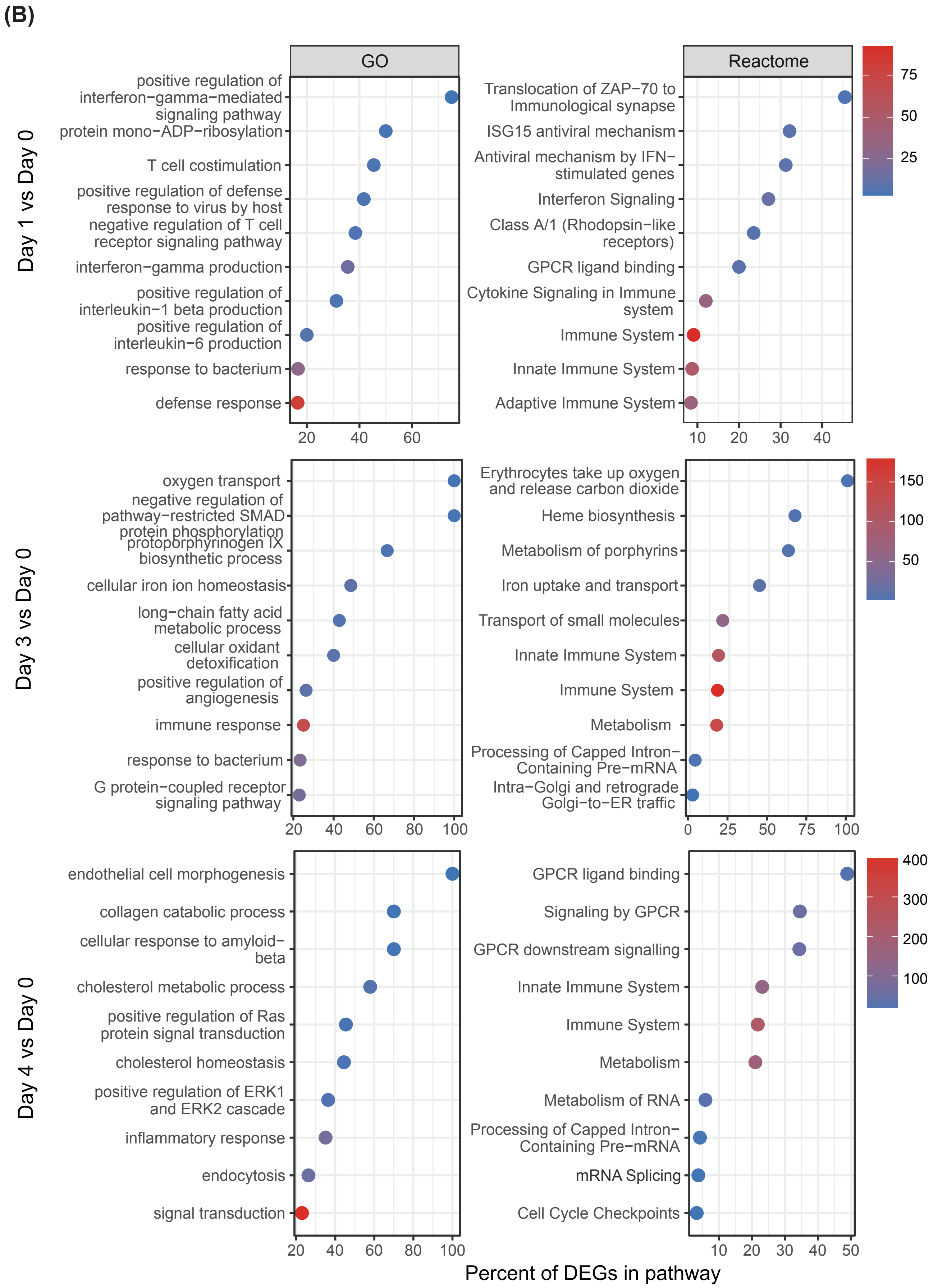
(B)
GO
Reactome
Day 1 vs Day 0
positive regulation of interferon−gamma−mediated signaling pathway
protein mono−ADP−ribosylation
T cell costimulation
positive regulation of defense response to virus by host
negative regulation of T cell receptor signaling pathway
interferon−gamma production
positive regulation of interleukin−1 beta production
positive regulation of interleukin−6 production
response to bacterium
defense response
Translocation of ZAP−70 to Immunological synapse
ISG15 antiviral mechanism
Antiviral mechanism by IFN−stimulated genes
Interferon Signaling
Class A/1 (Rhodopsin−like receptors)
GPCR ligand binding
Cytokine Signaling in Immune system
Immune System
Innate Immune System
Adaptive Immune System
Day 3 vs Day 0
oxygen transport
negative regulation of pathway−restricted SMAD protein phosphorylation
protoporphyrinogen IX biosynthetic process
cellular iron ion homeostasis
long−chain fatty acid metabolic process
cellular oxidant detoxification
positive regulation of angiogenesis
immune response
response to bacterium
G protein−coupled receptor signaling pathway
Erythrocytes take up oxygen and release carbon dioxide
Heme biosynthesis
Metabolism of porphyrins
Iron uptake and transport
Transport of small molecules
Innate Immune System
Immune System
Metabolism
Processing of Capped Intron−Containing Pre−mRNA
Intra−Golgi and retrograde Golgi−to−ER traffic
Day 4 vs Day 0
endothelial cell morphogenesis
collagen catabolic process
cellular response to amyloid−beta
cholesterol metabolic process
positive regulation of Ras protein signal transduction
cholesterol homeostasis
positive regulation of ERK1 and ERK2 cascade
inflammatory response
endocytosis
signal transduction
GPCR ligand binding
Signaling by GPCR
GPCR downstream signalling
Innate Immune System
Immune System
Metabolism
Metabolism of RNA
Processing of Capped Intron−Containing Pre−mRNA
mRNA Splicing
Cell Cycle Checkpoints
Percent of DEGs in pathway

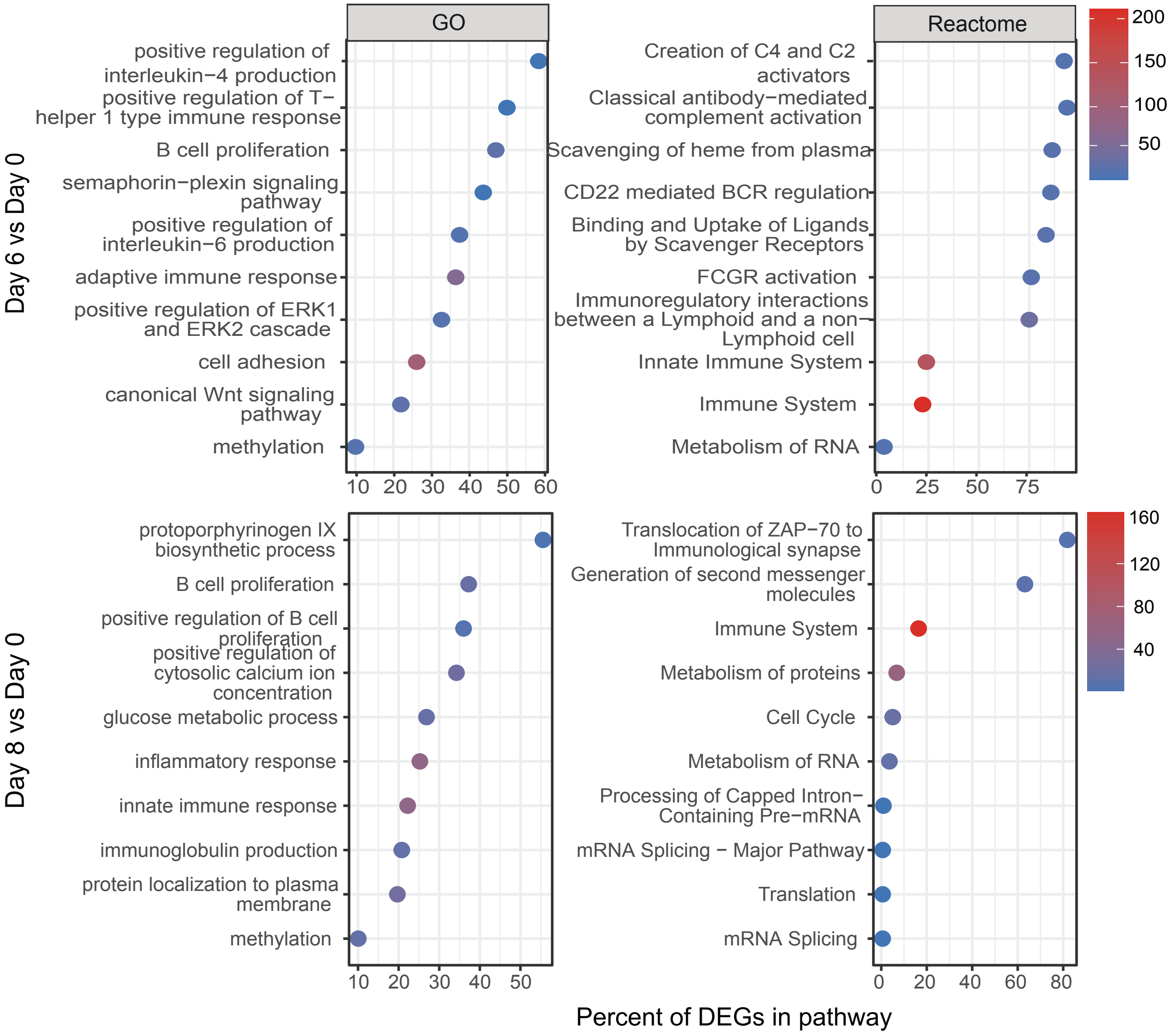

GO
Reactome
Day 6 vs Day 0
positive regulation of interleukin−4 production
positive regulation of T−helper 1 type immune response
B cell proliferation
semaphorin−plexin signaling pathway
positive regulation of interleukin−6 production
adaptive immune response
positive regulation of ERK1 and ERK2 cascade
cell adhesion
canonical Wnt signaling pathway
methylation
10 20 30 40 50 60
Creation of C4 and C2 activators
Classical antibody−mediated complement activation
Scavenging of heme from plasma
CD22 mediated BCR regulation
Binding and Uptake of Ligands by Scavenger Receptors
FCGR activation
Immunoregulatory interactions between a Lymphoid and a non−Lymphoid cell
Innate Immune System
Immune System
Metabolism of RNA
0 25 50 75
200
150
100
50
Day 8 vs Day 0
protoporphyrinogen IX biosynthetic process
B cell proliferation
positive regulation of B cell proliferation
positive regulation of cytosolic calcium ion concentration
glucose metabolic process
inflammatory response
innate immune response
immunoglobulin production
protein localization to plasma membrane
methylation
10 20 30 40 50
Translocation of ZAP−70 to Immunological synapse
Generation of second messenger molecules
Immune System
Metabolism of proteins
Cell Cycle
Metabolism of RNA
Processing of Capped Intron−Containing Pre−mRNA
mRNA Splicing − Major Pathway
Translation
mRNA Splicing
0 20 40 60 80
160
120
80
40
Percent of DEGs in pathway

Fig. S1. Top 10 GO and Reactome pathways by FDR on each day in the low (A) and high (B) inoculum cohorts.
The y-axis shows the pathway name, while the x-axis shows the percentage of genes out of the total number of genes in that pathway that were identified as differentially expressed on each respective day. Point color shows the absolute number of DEGs in the pathway on the corresponding day, with red representing a higher number of DEGs and blue a lower number. Note that color does not indicate up- or down-regulation of constituent genes in the pathway.
